# Filament deposition modeling quality assurance testing for the medical physicist

**DOI:** 10.1002/acm2.70856

**Published:** 2026-10-20

**Authors:** Benjamin T. Heggie, Manuel Arreola, Stephanie M. Leon, Izabella L. Barreto

**Affiliations:** ^1^ Department of Radiology University of Florida Gainesville Florida USA

**Keywords:** 3D printing, dual‐energy computed tomography, filament deposition modeling, quality assurance

## Abstract

**Background:**

Filament deposition modeling (FDM) can be a practical method for producing quality control (QC) imaging resources for the clinic. However, frequent printing may degrade print quality over time.

**Purpose:**

To develop a process to ensure prints for the diagnostic physicist are accurate and reproducible.

**Methods:**

Four FDM systems were evaluated by printing a custom, novel QC model on each printer 12 times over a period of 12 months. Several tests were performed, including print resolvability (lp/cm), XYZ dimension accuracy (mm), density (g/cm3), line width (mm), XY position accuracy (mm), clearance fits (mm), and thickness uniformity (mm). Polylactic acid (PLA) samples were printed, measured, and imaged with a dual energy CT scanner. Linear regression models were then created to monitor changes in QC density measurements as a function of x‐ray and material property characteristics (i.e., Hounsfield units and electron density). Between monthly prints, heat uniformity across the build plate was also tested for each printer using a NIST‐certified infrared thermometer and 3D printed grids. All physical measurements were obtained using a calibrated digital caliper and milligram scale.

**Results:**

Mean percent error (MPE) for all tests was calculated from ground truth (CAD dimensions and filament density) and baseline values (first month of QC measurements). Among printers investigated, MPE was ± 5% of ground truth for all tests except density and thickness uniformity. Baseline MPE was also observed within ± 5% except for resolvability and clearance fit tests among printers. Subtle trend increases were presented for higher density QC prints based on DECT linear regression models. Reduced chi‐squared values indicate uniform and reproducible heat bed temperature measurements.

**Conclusions:**

This work offers an innovative approach toward evaluating and monitoring FDM system performance. Future work should track performance over a longer time period and across a wider variety of printers.

## 1 INTRODUCTION

3D printing has become a resource for producing various tools useful in radiological imaging, including quality control (QC) equipment tailored to the needs of the diagnostic physicist.^1^, ^2^, ^3^, ^4^ Some researchers have suggested that the need to purchase commercial phantoms for clinical medical physics QA procedures may eventually be diminished.^5^, ^6^ Studies have characterized a variety of composite printing materials and slicer software techniques to develop phantoms with tissue‐equivalent structures ^7^, ^8^, ^9^, ^10^, ^11^, ^12^, ^13^ or for medical physics test equipment.^14^ Despite the recognized potential, a systematic QC process is needed to evaluate and monitor the accuracy and reproducibility of 3D printing systems. As imaging modalities undergo routine quality assurance (QA) to ensure consistent performance, 3D printer performance may also drift over time, resulting in prints with inaccurate dimensions, nonuniformities, or printing artifacts such as shrinkage, stringing, or warping.^15^, ^16^, ^17^ Consequently, phantoms reprinted over time using identical polymers, printing files, and scanning procedures may not yield consistent or accurate imaging results, presenting a need for dedicated QC models for the 3D printers themselves.

Recommended quality standards for 3D printers should be based on clinical performance characteristics that imaging phantoms are expected to evaluate, such as geometric accuracy, x‐ray attenuation characteristics, spatial resolution, and low‐contrast detectability. To be clinically useful, printed phantoms must therefore reproduce these features with sufficient dimensional and material accuracy. This can be challenging for 3D printers due to limitations in printable materials available across technologies, model construction, and differences in performance among additive manufacturing (AM) processes. While various AM processes are available, including selective laser sintering (SLS), stereolithography (SLA), material jetting (MJ), or filament deposition modeling (FDM),^18^, ^19^, ^20^ this work focuses on FDM printers since they include adjustable infill patterns, multi‐extrusion capabilities, affordability, feasibility, and diversity in build volumes, filament materials, and spool capacities.^21^, ^22^ FDM printers also typically require only basic ventilation and minimal safety equipment, reducing the need for specialized lab spaces.^23^, ^24^ However, specific printer characteristics and calibration techniques applied by different vendors can influence QA phantom production, resulting in a need to test multiple FDM printers.

Use of FDM for precision phantoms requires a detailed understanding of FDM printing concepts and slicer functions responsible for targeting desired imaging measurements. For instance, flow rate, which governs the rate and line width of filament extruded per layer, depends on melting temperature, printing speed, and flow percentage.^25^, ^26^, ^27^ Changes in flow rate from declines in printer performance can be detected in changes in object density as well as x‐ray and material property imaging maps.^28^, ^29^ Other slicer functions, such as infill density and infill pattern, allow users to produce prints with more or less extruded lines of filament per layer.^10^, ^12^ Hence, diverse infill options should also be investigated to monitor corresponding changes in density and imaging measurements for printed objects.

This work introduces a novel approach for testing FDM systems using calibrated equipment and a dual‐energy computed tomography (DECT) scanner. Unlike most studies, rather than relying on segmented anatomical models, the QC model in this work is designed with CAD software, providing more control over what is tested and minimizing detail loss during g‐code file conversions.^30^ In addition to assessing resolvability and XYZ dimensional accuracy, the model incorporates positional accuracy tests based on typical FDM resolution (0.1–0.4 mm for XY dimensions and 0.1–0.5 for Z dimensions).^31^ Clearance‐fit testing is also included to establish recommended tolerances for different FDM printers to produce phantom assemblies.^32^ Infill density and flow settings commonly used to 3D print tissue equivalent materials or contrast agents are tested over time to monitor reproducibility in density measurements as a function of imaging metrics. Additional methods for assessing build‐plate conditions such as leveling or heat uniformity are also introduced.^33^ DECT was used to monitor print quality because it provides quantitative information that is relevant to evaluating materials used in phantom construction. Mass attenuation coefficients are defined at monoenergetic energies and form the basis for calculating reference CT number values for clinically relevant materials. Virtual monoenergetic images (VMI) approximate these monoenergetic conditions and are therefore suited for assessing whether printed structures achieve the expected attenuation properties. Prior work has shown that VMI reconstructions are just as reproducible as conventional CT images,^34^ supporting their use for QC measurements. DECT also enables assessment of whether printed materials are reproducible enough for applications that rely on accurate electron density or effective atomic number measurements, such as in radiation therapy planning.^35^

The purpose of this study is to create a filament deposition modeling quality control (FDMQC) model and testing process that medical physicists can use to evaluate and monitor 3D printer performance. This work also investigates multiple FDM printers from a variety of vendors to assess differences in accuracy and reproducibility measurements.

## 2 METHODS

### 2.1 3D printers, filaments, & measuring instruments

Four FDM printers were investigated in this work: the UltiMaker S7 (UltiMaker, Utrecht, Netherlands), QIDI Tech X‐Max 3 (QIDI Technology, Ruian, Zhejiang Province, China), Original Prusa MK4 (Prusa Research, Prague, Czech Republic), and Bambu Lab A1 Mini (Bambu Lab, Shenzhen, China). Custom 3D printed grids and a NIST‐certified infrared thermometer (Triplett Test Equipment & Tools, Manchester, NH, USA) were used to investigate heat bed uniformity for each printer. A NIST‐certified digital caliper (Control Company, Webster, TX, USA) and a MAXUS digital milligram scale (Starsea Limited) were used to perform physical measurements on printed QC models. A NIST‐certified weight set (Troemner LLC, Thorofare, NJ, USA) was also used to validate the accuracy of the milligram scale. To avoid measurement bias consistent with mass or composition differences in filament batches, each printer was equipped with a 3‐kg batch of PolyLite PLA (Polymaker, Changshu, China), which was sufficient to print all QC models in this work. Specifications for FDM printers and PolyLite batches are listed in Table 1.

**TABLE 1:** Specifications for FDM printers and PolyLite PLA filament.

Specifications	UltiMaker S7	QIDI X‐Max 3	Original Prusa MK4	Bambu A1 Mini
Build volume (mm)	330×240×300	325×325 x 315	250×210 x 220	180×180×180
Extruder type	Bowden	Direct	Direct	Direct
Nozzle diameter (mm)	0.4	0.4	0.4	0.4
Filament diameter (mm)	2.85	1.75	1.75	1.75
Filament color	White	Blue	Red	Grey
Filament SKU #	PA02038	PA02067	PA02066	PA02035

Note: Stock Keeping Unit number (SKU #) tracks filaments by name and color

### 2.2 Heat bed uniformity testing

Prior to printing the QC model, heat uniformity across build plates was evaluated. First, the heat bed for each printer was set to 60 degrees Celsius for approximately 10 minutes. Next, an infrared thermometer was used to measure temperature in each 50×50 mm cell within custom 3D‐printed heat bed grids, each with a matrix size covering the print field area; (UltiMaker S7: 6×5, QIDI X‐Max 3: 6×6, Prusa MK4: 5×4, Bambu A1 Mini: 3×3). Under ambient room conditions (22–24°C), all measurements were performed indoors in an office environment. Reduced chi‐square values were then calculated for each set of measurements.

### 2.3 Designing, printing, and measuring a quality control model for FDM printers

A quality control model (Figure 1A) was custom designed for this work with SolidWorks CAD modeling software (Student Edition 2025 SP4.0, Dassault Systèmes, Vèlizy‐Villacoublay, France) consisting of various objects for FDM performance assessment, including twelve cylinders for measuring density (g/cm^3^), a peg and hole assembly for determining clearance fitting tolerances, a cube for measuring line width, and a five‐tier pyramid for measuring Z‐dimensions. These objects were positioned inside of a thin base plate that includes QC testing features such as bar patterns for resolvability assessment, peg and hole diameters for measuring XY dimensions, and landmarks for measuring XY positions. The CAD files for each object were converted to STL files, representing 3D model surfaces as a triangular mesh. Each STL file was uploaded into slicer software compatible with each printer (Table 2), and print settings were selected based on recommendations from the slicer software and filament manufacturer.

**FIGURE 1:**
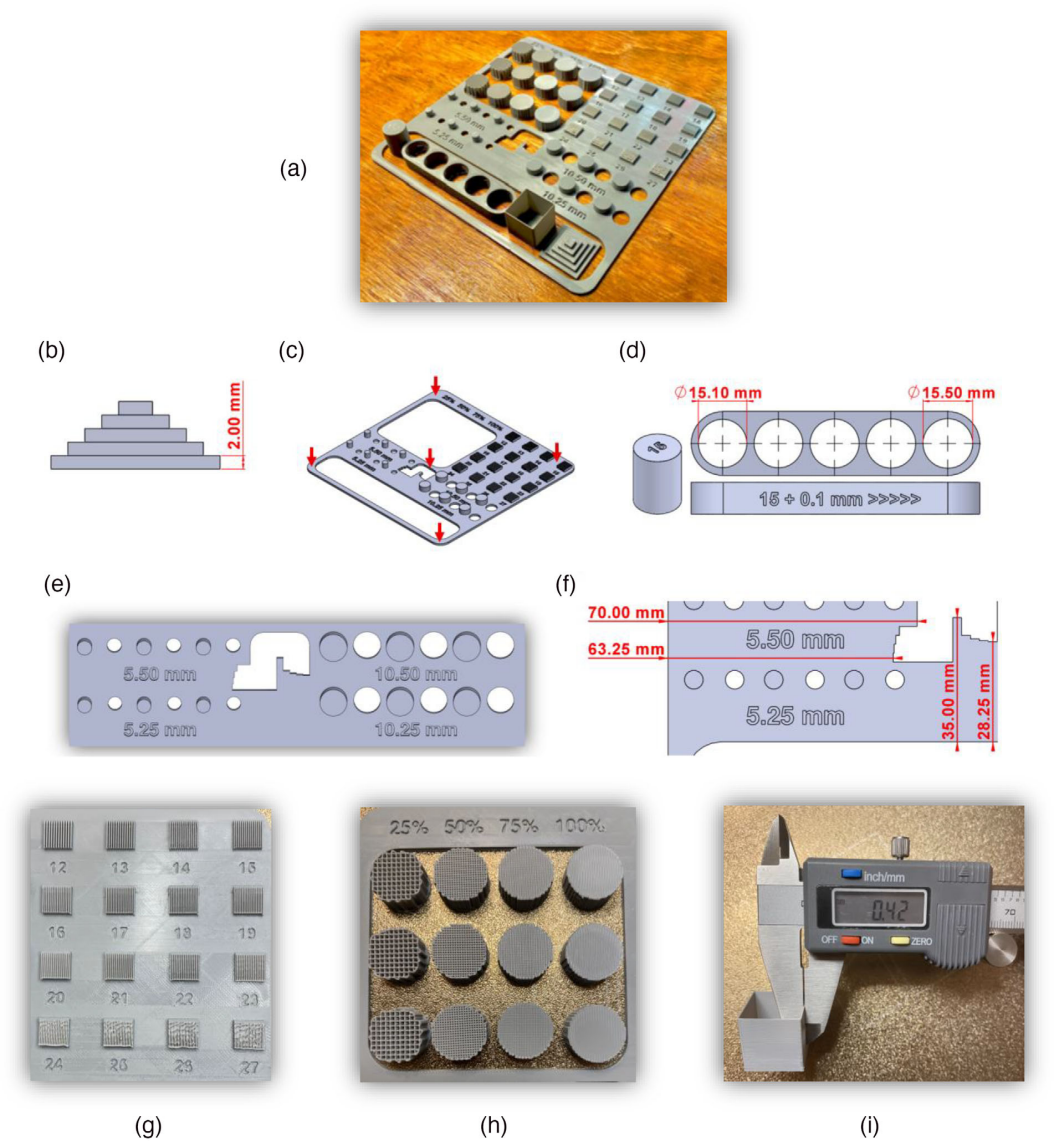
FDMQC model and QC procedures evaluated. (A) Complete FDMQC model printed with Bambu A1 Mini; (B) Z‐dimension accuracy with 5‐level pyramid; (C) base‐plate uniformity thickness with 5 measurement zones shown in red; (D) clearance fit reproducibility with 15‐mm peg and holes of 15.10, 15.20, 15.30, 15.40, 15.50 mm; (E) XY‐dimension accuracy consisting of three pegs & holes per set of diameters: 5.25, 5.50, 10.25, and 10.50 mm; (F) XY‐position accuracy with distances in X of: 63.25, 63.50, 64.00, 65.00, & 70.00 mm and Y of: 28.25, 28.50, 29.00, 30.00, & 35.00 mm; (G) qualitatively‐resolved bar pattern groups (12‐27 lp/cm); (H) density (g/cm^3^) for four sets of three cylinders, each printed with separate infill density settings of 25, 50, 75, & 100%; and (I) line width (mm) for an open‐top cube

**TABLE 2:** FDM slicer software and applied print settings for QC models printed

Print settings	UltiMaker cura	QIDISlicer	PrusaSlicer	Bambu studio
Melting temperature (C)	210	210	220	220
Build plate temperature (C)	60	60	60	60
Printing speed (mm/s)	60	100	100	100
Flow percentage (%)	82.35	95.45	102.4	100

Additional print settings were defined for each QC test illustrated in Figure 1B‐I. The QC model components intended for evaluating Z‐dimension (Figure 1B) and thickness uniformity (Figure 1C) were printed using an infill layer height of 0.20 mm to target print dimensions as specified in the CAD model. Caliper measurements were performed for both QC tests to determine the average pyramid layer thickness (2 mm thick) for Z‐dimension assessment and average base plate thickness for uniformity assessment. Clearance fitting (Figure 1D) tests were conducted by manually feeding a peg through five holes from largest to smallest diameter until a desired clearance fit with little‐to‐no resistance was achieved.

For XY dimension testing (Figure 1E), four sets of peg and hole diameters were measured with calipers and averaged for each set consisting of three pegs and holes. XY‐position measurements (Figure 1F), were also determined with calipers using five center‐to‐edge distances, defined in the baseplate CAD model, across X‐ and Y‐directions. Resolvability (Figure 1G) was evaluated qualitatively by identifying the smallest resolvable bar pattern (12‐27 lp/cm) without PLA intersections present. For density measurements (Figure 1H), four sets of three cylinders were printed with infill density settings of 25%, 50%, 75%, and 100%. The average density of each set was calculated by dividing the measured average mass by the volume defined in the CAD file (∼2.5 cm^3^). Finally, line width (Figure 1I) was determined by measuring the walls of an open‐top hollow cube printed with 0% infill density, zero top layers, one wall, and a nominal line width of 0.42 mm.

Before printing an FDMQC phantom, flow settings were calibrated for each printer to extrude a line width of 0.42 mm, which approximates the nozzle diameter. A hollow open‐top cube was printed at 100% flow and calipers were used to measure the average wall thickness. The corrected flow setting ($Flow_{c}$) that will produce the corrected line width (0.42 mm) was calculated using Equation 1.

(1) $$Flo\;w_{C}=\frac{Targeted\;Line\;Width\;\left(0.42\;mm\right)}{Initial\;Line\;Width}\;\times 100\%\;Flow$$

Corrected flow settings listed in Table 2 were then reapplied for future QC models printed to monitor any changes in line width over time. The printing instructions in g‐code files for each printer were then created and uploaded for printing.

The QC model was printed on each printer once a month for twelve months. Each printer accumulated at least 20 hours of monthly print time prior to printing a QC model (range: 20–288 hours). For each print, physical and qualitative measurements were performed as described above.

### 2.4 Calculated error in quality control measurements

Based on the QC measurements described previously, the mean percent error (MPE) was calculated using (1) CAD model dimensions and density for PolyLite PLA (1.17 g/cm^3^) to determine the accuracy of FDM printers and (2) the first month of QC measurements as baseline values to monitor the reproducibility of FDM prints.

### 2.5 Estimating DECT measurements from density for PolyLite PLA samples

Rather than repeatedly using a CT scanner to assess samples over time, linear regression models were created and used to calculate corresponding DECT imaging values for density measurements determined from the QC models. This allows separation from drifts in CT scanner performance over time, reduces the need to access the CT scanner multiple times, and evaluates FDM print reproducibility in terms of imaging values. Therefore, this step is meant to serve as a feasible approach for the physicist to assess temporal changes in print quality.

To generate the linear regression model, a commercial water‐equivalent multi‐energy CT head phantom (Model 1472, Sun Nuclear, Melbourne, Florida) was loaded with 3D‐printed samples of PolyLite PLA and scanned with a DECT scanner (Aquilion One Prism, Canon Medical Systems Corporation, Otawara, Japan) using rapid‐kV switching (RKS) and deep‐learning‐based spectral image reconstruction. Twelve samples were printed from each printer using different infill density (25, 50, 75, 100%) and flow (95, 100, 105%) combinations. The density of each was determined by dividing mass measurements recorded from a milligram scale with the volume defined in the CAD file (13.7 cm^3^). Samples were then loaded into groups of three along the phantom periphery, where each group had samples from the same printer, with the same infill density, but different flow settings. The phantom was centered at isocenter, scanned with imaging parameters defined in Table 3, and measured with image processing software (Vitrea Advanced Visualization, Canon Medical Informatics, Minnetonka, Minnesota) to record CT number from VMIs at 60 keV and electron density. Scanner units for electron density ($10^{23}\cdot e/cm^{3}$) were converted to ($e/cm^{3}$) and expressed relative to water. This entire process was repeated three times to calculate the error range and standard error of the mean (SEM) for each printer. Separate linear regression models were generated for CT number and relative electron density as functions of measured print density. These models were developed solely to estimate expected imaging values from measured print densities and not to model the relationship between CT number and electron density.

**TABLE 3:** DECT scanning parameters

Scan parameters	
Scan mode	Helical RKS
kVp	135 & 80
mA	600
Slice thickness (mm)	1
Rotation tme (s)	0.50
Pitch	0.813
Reconstruction	Spectral

Using the linear regression models, DECT metrics were calculated and plotted for each printer based on average density measurements recorded for the monthly QC models printed.

## 3 RESULTS

### 3.1 Heat bed uniformity

Reduced chi‐squared values calculated from monthly heat bed temperature data sets ranged from 0.16–0.45 for the Ultimaker S7, 0.13–0.66 for the QIDI X‐Max 3, 0.55–1.0 for the Original Prusa MK4, and 0.09–0.67 for the Bambu A1 Mini. Each set of ranges indicated no significant variation in the observed distribution in heat across build plates. On average, the measured bed temperature for each printer deviated from the set temperature (60 C°) by 1.0C°–4.0 C°, except for the Original Prusa, which deviated by 9.0 C°.

### 3.2 Accuracy in FDMQC measurements

Across all printers, the MPE for the XY dimension measurements ranged from −4.9% to −0.25%. Among all QC tests, MPE ranges were best for Z‐dimension accuracy (−0.23 to +2.0%), line width accuracy (−1.1 to +1.6%), and position accuracy (−0.72 to +0.30%). The largest deviations from ground truth were observed for density (−20.98% to +28.92%) and base plate thickness uniformity (+7.34% to +11.43%). Clearance fits were more accurate for the Ultimaker S7 (0.10 mm) than the other printers (0.20–0.40 mm). Calculated MPE values relative to ground truth for each printer and quantitative QC test are shown in Table 4.

**TABLE 4:** Mean percent error relative to ground truth values defined in the CAD model for each FDM Printer and QC test. Positive values indicate measurements above ground truth; negative values indicate measurements below ground truth.

QC Test	Setting	Ultimaker S7 MPE (%)	QIDI X‐Max 3 MPE (%)	Original Prusa MK4 MPE (%)	Bambu A1 Mini MPE (%)
**Density**	25% infill	21.31	27.21	26.95	28.92
	50% infill	0.43	12.46	11.40	14.21
	75% infill	−10.16	5.57	4.88	7.45
	100% infill	−20.98	0.52	−0.60	2.48
**Line width**	0.42 mm	−1.14	0.30	1.59	0.89
**Z‐dimension**	2 mm	−0.23	2.03	0.62	0.97
**Thickness uniformity**	Base plate	9.68	11.43	7.34	9.30
**Peg XY‐dimension**	10.50 mm	−2.07	−0.68	−1.21	−1.59
	10.25 mm	−2.33	−0.70	−0.98	−1.54
	5.50 mm	−4.93	−0.25	−1.76	−1.93
	5.25 mm	−4.58	−0.33	−1.41	−1.99
**Hole XY dimension**	10.50 mm	−0.70	−1.22	−1.32	−1.20
	10.25 mm	−0.75	−1.21	−1.34	−1.14
	5.50 mm	−3.06	−2.86	−3.83	−3.68
	5.25 mm	−3.23	−2.03	−3.81	−3.35
**X‐position**	70 mm	−0.22	−0.04	−0.06	−0.33
	65 mm	−0.32	−0.07	−0.06	−0.34
	64 mm	−0.23	−0.06	−0.07	−0.36
	63.50 mm	−0.23	−0.05	−0.03	−0.35
	63.25 mm	−0.18	−0.03	0.00	−0.37
**Y‐position**	35 mm	−0.51	0.24	0.29	0.05
	30 mm	−0.67	0.30	0.30	0.09
	29 mm	−0.72	0.19	0.26	−0.01
	28.50 mm	−0.67	0.18	0.17	0.04
	28.25 mm	−0.43	0.25	0.15	0.09

### 3.3 Reproducibility in FDMQC measurements

Across all printers, resolvability measurements ranged from 15–24 lp/cm, with baseline MPE highest for the QIDI X‐Max 3 at 31% and lowest for the Bambu A1 Mini at 3.6%. The QIDI printer also showed a higher baseline MPE range for XY dimensions (+0.45 to +4.5%) than all other printers. The lowest MPE range was observed for Z‐dimension (−1.7 to 0.36%) and position accuracy (−0.26 to 0.33%) QC tests. Clearance fits were extremely reproducible for the Ultimaker S7 where the recommended fit between the peg and smallest hole diameter (0.10 mm) was recorded for all months.

### 3.4 Reproducibility for scanned phantom samples

Reproducibility in imaging and density measurements was evaluated for the rod samples that were scanned. Among the 12 data points for infill densities investigated, the SEM for CT number measurements at 60 keV ranged the highest for the Ultimaker S7 (3.1×10^−1^ to 1.5×10^0^) and lowest for the Bambu A1 Mini (1.6×10^−1^ to 7.9×10^−1^). For electron density data points, the SEM also ranged the highest for the Ultimaker S7 (6.7×10^−4^ to 3.7×10^−3^) and lowest for the Bambu A1 Mini (5.3×10^−4^ to 2.5×10^−3^). The maximum and minimum SEM observed for density measurements determined among printers was for the Ultimaker S7 (5.9×10^−3^) and QIDI X‐Max 3 (4.2×10^−5^), respectively. Linear regression models relating measured print density to CT number and relative electron density were significant for all printers (p < 0.001 for all fits).

The potential deviations in FDMQC VMI and relative electron density measurements that would be a consequence of variations in reproducibility were determined by calculating the change in measured density of the cylinders from baseline for each QC model and then using the linear regression models to extrapolate corresponding values. The mean and range of calculated CT number at 60 keV and relative electron density across the 12 monthly FDMQC models are summarized for each printer and infill density setting in Table 5. The range in error for calculated CT number values at 60 keV was determined for each printer; Ultimaker S7: (−11 to 40 HU), QIDI X‐Max 3: (−9 to 32 HU), Bambu A1 Mini: (−12 to 16 HU), and Prusa MK4 (−13 to 20 HU). The range in error was also determined for the calculated electron density values; Ultimaker S7: (−1.2×10^−2^ to 4.2×10^−2^), QIDI X‐Max 3: (−9.1×10^−3^ to 3.3×10^−2^), Bambu A1 Mini: (−9.9×10^−3^ to 1.9×10^−2^), and Prusa MK4: (−1.4×10^−2^ to 2.1×10^−2^).

**TABLE 5:** Mean and range of calculated CT number at 60 keV and relative electron density across 12 monthly FDMQC models for each printer and infill density setting.

Printer	Infill density	CT number at 60 keV, mean (range)	Relative electron density, mean (range)
Ultimaker S7	25%	−680.0 (−688.3 to −673.5)	0.309 (0.300 to 0.316)
	50%	−464.2 (−478.2 to −455.1)	0.533 (0.519 to 0.543)
	75%	−255.1 (−271.7 to −241.2)	0.751 (0.733 to 0.765)
	100%	−84.5 (−107.5 to −67.1)	0.928 (0.904 to 0.946)
QIDI X‐Max 3	25%	−632.9 (−638.6 to −627.5)	0.358 (0.352 to 0.363)
	50%	−376.4 (−386.6 to −367.1)	0.626 (0.615 to 0.636)
	75%	−125.2 (−137.7 to −110.0)	0.889 (0.876 to 0.905)
	100%	112.3 (93.8 to 134.1)	1.137 (1.118 to 1.160)
Original Prusa MK4	25%	−637.6 (−640.7 to −632.4)	0.359 (0.355 to 0.364)
	50%	−387.6 (−393.9 to −378.2)	0.622 (0.615 to 0.632)
	75%	−135.3 (−153.2 to −120.1)	0.887 (0.868 to 0.903)
	100%	96.0 (78.5 to 109.8)	1.131 (1.112 to 1.145)
Bambu A1 Mini	25%	−624.4 (−628.7 to −617.9)	0.373 (0.369 to 0.380)
	50%	−359.5 (−365.1 to −346.0)	0.648 (0.642 to 0.662)
	75%	−98.7 (−104.2 to −87.1)	0.919 (0.913 to 0.931)
	100%	147.9 (138.2 to 156.7)	1.175 (1.165 to 1.184)

## 4 DISCUSSION

Reduced chi‐square values determined from each printer indicate strong heat uniformity across the build plate and no signs of degradation over a 12‐month period. The offset observed for the Original Prusa MK4 suggests poor calibration and that higher bed temperatures be used to promote the bond between prints and the build plate, especially for polymers that require higher bed temperatures, such as Acrylonitrile Butadiene Styrene (ABS) and Acrylonitrile Styrene Acrylate (ASA).

Based on the calculated error (dimensional & baseline) determined among printers for FDMQC measurements, tests for evaluating line width, Z‐dimensions, XY dimensions, and XY positions were accurate, reproducible, and within the typical tolerance range (0.10–0.50 mm) mentioned for FDM printers.^31^ Resolvability assessments indicate subtle differences in system performance among printers, making them reliable for printing high contrast imaging tools limited to 19–21 lp/cm for a 0.40 mm extruder nozzle. Although this range is acceptable to evaluate typical resolutions, for example CT protocols (6–7 lp/cm for soft tissue or 8.0–10 lp/cm for bone reconstructions), it is not small enough to evaluate modalities that demand sharper resolvability assessment such as digital mammography systems (∼5.0–10 lp/mm).^36^, ^37^ The density measurements indicate no signs of degradation over time; however, compared to the other printers, the Ultimaker S7 showed higher error values for cylinders printed with higher infill density settings, which may reflect inconsistent flow issues in Bowden systems compared to direct drive systems.^38^ Most studies demonstrated a similar geometric error within 1 mm using printed anatomical models or phantoms.^39^, ^40^, ^41^ However, this may not always be the case for all printer models and vendors, based on the results of a study which investigated 16 printers by printing a QA phantom to test dimensional accuracy and precision of various shapes and anatomical structures.^42^

Printer performance varied from baseline for resolvability (MPE: 13% for Ultimaker S7, 31% for QIDI Tech X‐Max 3, 12% for Original Prusa MK4, and 3.6% for Bambu A1 Mini). The Ultimaker S7 showed consistent fits for the smallest hole diameter every month (0.10 mm, MPE ∼0%). The other printers, however, ranged between 0.20–0.30 mm while presenting some drifting in baseline MPE of up to 0.40 mm for the QIDI printer. Recommended tolerances should be different for each printer when making assembly models: 0.10 mm for Ultimaker, 0.25 mm for the Prusa and Bambu printers, and 0.40 mm for QIDI. Since fit can change over time, a QC model should be printed and assessed prior to printing complex models.

While the fluctuations observed in monthly calculated HU and electron density values determined among printers may worsen due to degradation or frequent use, a potential cause for the deviations observed in this work is printer performance. This notion is best supported when comparing the SEM and baseline error range for the Ultimaker S7 (Bowden system) to the other printers (direct drive systems). Consequently, direct drive systems may be better suited for the fabrication of imaging phantoms. Additionally, the Bambu A1 Mini demonstrated greater measurement reproducibility compared to the other printers; it uses an automated flow dynamic calibration technique, promoting print uniformity. Other automated calibration features that promote print uniformity should also be considered when selecting 3D printers for phantom fabrication. Another possibility for the fluctuation is variation in the performance of the CT scanner, but this seems unlikely to be the primary cause because the changes in CT number and electron density values do not appear to be correlated among printers.

The linear regression models used to estimate CT number and relative electron density from measured print density serve two purposes. First, changes in measured print density during routine QC can be translated into expected changes in imaging values, allowing physicists to monitor changes in printer performance over time without repeatedly scanning QC models. Second, the models may be used for cross‐calibration among FDM printers by selecting a standardized imaging value, such as CT number at 60 keV, and determining the corresponding print density required for each printer. Printer‐specific parameters, such as infill density or flow, can then be adjusted to target the required print density, potentially allowing different FDM printers to produce phantoms with comparable attenuation properties.

This study has limitations. First, complex anatomical structures were not assessed. However, the simpler objects in the QC model are standardized in a way that more easily translates among printers, allowing simple customization of values relevant to other printers, and troubleshooting of specific printer flaws. Furthermore, this work is also limited to the investigation of FDM printers, the specific printers investigated, and the number of printing hours accumulated each month between QC models. Future studies should develop QC methods specific to other 3D printing technologies used for developing quality phantoms. For example, resin printers use settings such as cure time (s), light intensity (%), and lift speed (mm/s), all of which should be explored to assess potential influences, if any, on x‐ray attenuation characteristics and material properties of prints. While the QC model introduced in this work is intended for the assessment of FDM printers, geometric and resolvability components (base plate, clearance fitting, and pyramid) may be printed and used to evaluate other 3D printing systems, except those meant to assess FDM techniques such as infill density (cylinders) and flow (cube). Other DECT images such as virtual non‐contrast, iodine maps, or effective atomic number are also not considered since the PLA in this work is low‐density material. Studies have also demonstrated issues targeting accurate effective atomic number measurements for low‐density composites such as lung tissue, foregoing the use of accurate linear regression models, as applied in this work, to monitor FDM performance.^43^, ^44^, ^45^ Lastly, while this work used the same spools to produce QC models for each printer, utilizing new filament batches with variations in manufactured filament diameter or composition may yield differences in QC measurements, requiring additional validation between filament batches.^46^

The tolerances established in this work for FDM printers should not be assumed to apply directly to other printing technologies. When adapting these QC methods to another technology, cross‐over testing should first be performed by printing the geometric and resolvability components introduced from this work, followed by a measurement comparison against the same CAD‐defined ground‐truth values. Technology‐specific baseline measurements and acceptance tolerances could then be established before the process is used for routine QC.

## 5 CONCLUSION

FDM printers are accurate and reproducible based on the QC process conducted. Results from applied testing methods indicate consistency in measurements from both a physical and imaging perspective. Custom designed QC models and processes are an effective method for the assessment of FDM performance and development of quality phantoms for imaging.

## AUTHOR CONTRIBUTIONS

BH participated in the study design, data collection, data analysis, and writing of the manuscript. MA participated in conceptualization, supervision, and providing resources and funding. SL and IB participated in study design, data analysis, and manuscript revision. All authors approved the final version of the manuscript.

## CONFLICT OF INTEREST STATEMENT

The authors declare no conflicts of interest.

## Supporting information




Supporting Information

Supporting Information

Supporting Information

Supporting Information

Supporting Information

Supporting Information

Supporting Information

Supporting Information

Supporting Information

Supporting Information

Supporting Information

Supporting Information

## Data Availability

The data that support the findings of this study are available from the corresponding author upon reasonable request. The STL and CAD files comprising the FDMQC model introduced in this work are available as supplementary material.
